# Calming effect of Clinically Designed Improvisatory Music for patients admitted to the epilepsy monitoring unit during the COVID-19 pandemic: a pilot study

**DOI:** 10.3389/fneur.2023.1206171

**Published:** 2023-09-01

**Authors:** Borna Bonakdarpour, Guangyu Zhou, Daniel Huang, Catherine T. Vidano, Stephan Schuele, Christina Zelano, Clara Takarabe

**Affiliations:** ^1^Department of Neurology, Feinberg School of Medicine, Northwestern University, Chicago, IL, United States; ^2^Feinberg School of Medicine, Mesulam Center for Cognitive Neurology and Alzheimer’s Disease, Northwestern University, Chicago, IL, United States

**Keywords:** music intervention, COVID-19, epilepsy monitoring unit, quality of care, improvisation (music)

## Abstract

**Background:**

Epilepsy monitoring requires simulating seizure-inducing conditions which frequently causes discomfort to epilepsy monitoring unit (EMU) patients. COVID-19 hospital restrictions added another layer of stress during hospital admissions. The purpose of this pilot study was to provide evidence that live virtual Clinically Designed Improvisatory Music (CDIM) brings relief to EMU patients for their psychological distress.

**Methods:**

Five persons with epilepsy (PWEs) in the EMU during the COVID-19 lockdown participated in the study (average age ± SD = 30.2 ± 6 years). Continuous electroencephalogram (EEG) and electrocardiogram (EKG) were obtained before, during, and after live virtual CDIM. CDIM consisted of 40 minutes of calming music played by a certified clinical music practitioner (CMP) on viola. Post-intervention surveys assessed patients’ emotional state on a 1–10 Likert scale. Alpha/beta power spectral density ratio was calculated for each subject across the brain and was evaluated using one-way repeated analysis of variance, comparing 20 minutes before, during, and 20 minutes after CDIM. Post-hoc analysis was performed using paired *t*-test at the whole brain level and regions with peak changes.

**Results:**

Patients reported enhanced emotional state (9 ± 1.26), decrease in tension (9.6 ± 0.49), decreased restlessness (8.6 ± 0.80), increased pleasure (9.2 ± 0.98), and likelihood to recommend (10 ± 0) on a 10-point Likert scale. Based on one-way repeated analysis of variance, alpha/beta ratio increased at whole-brain analysis (F_3,12_ = 5.01, *P* = 0.018) with a peak in midline (F_3,12_ = 6.63, *P* = 0.0068 for Cz) and anterior medial frontal region (F_3,12_ = 6.45, *P* = 0.0076 for Fz) during CDIM and showed a trend to remain increased post-intervention.

**Conclusion:**

In this pilot study, we found positive effects of CDIM as reported by patients, and an increased alpha/beta ratio with meaningful electroencephalographic correlates due to the calming effects in response to CDIM. Our study provides proof of concept that live virtual CDIM offered demonstrable comfort with biologic correlations for patients admitted in the EMU during the COVID-19 pandemic.

## Background

### Functional neuroanatomy of music processing

**Figure 1:**
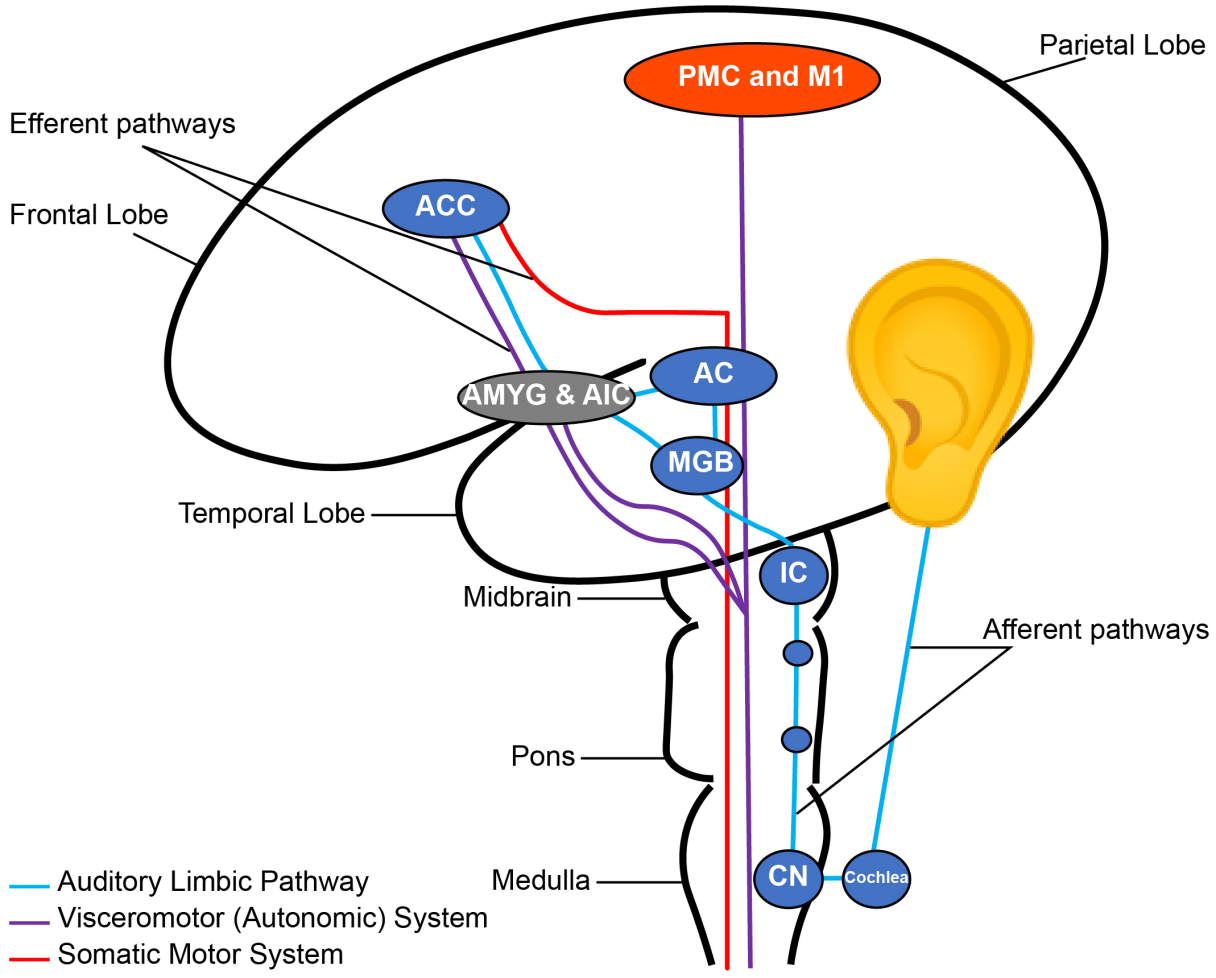
Nodes of the auditory pathway. CN, Cochlear Nuclei; IC, Inferior Colliculus; MGB, Medial Geniculate Body; AC, Auditory cortex; AMYG, Amygdala; AIC, Anterior Insular Cortex; ACC, Anterior Cingulate Cortex; PMC, Primary Motor Cortex; M1, Primary Motor Cortex. Note the connection between the auditory and emotional regions through the auditory limbic pathway. Reconstructed from Koelsch (2).

Music has an extensive effect on the human brain from early brain stem auditory processing to the most complex mechanisms of musical perception, recognition, emotion, and interaction with other brain networks (1, 2) (Figure 1). Heschl’s gyrus, parts of the planum temporale, and the posterior superior temporal gyrus are located in the posterior section of the superior temporal lobe; these constitute the auditory cortex. The primary auditory cortex in Heschl’s gyrus is required for decoding a musical stimulus and is strongly implicated in perceiving tone (3). However, there are several regions beyond the Heschl’s gyrus that are crucial to the perception of music. After the Heschl’s gyrus receives information from the medial geniculate nucleus, it is postulated to make network connections to the auditory association cortex (most sensitive to perceiving pitch and performing complex musical processing tasks), mesolimbic systems, and other multi-sensory cortices (4). These areas are similar to those also activated in speech. For rhythm, there is more activation in the basal ganglia and cerebellum, implicating a motor component to rhythmic listening. Recognizing music and producing an emotional response are thought to localize in the limbic system and orbitofrontal areas via the planum polare (5). Additionally, musicians have been shown to possess structural differences in a variety of regions including auditory (6), motor (7), callosal (8), somatosensory, superior parietal (9), and cerebellar (10) reflecting other determinants of musical perception. This distributed effect of music on the brain has made it a non-pharmacological candidate for its effects on brain functions.

### Neurobiology of rhythmic entrainment

One of the major innate responses to music is through entrainment. Entrainment is defined by a temporal locking process in which one system’s motion or signal frequency phase-locks the frequency of another system (11). The auditory system has richly distributed fiber connections to motor centers from the spinal cord upward to the brain stem, cerebellum, basal ganglia, and motor cortex. The effects of music on reticulospinal pathways was suggested as early as 1960s and 1970s (12, 13). Through entrainment, music not only induces physical rhythmic movements such as tapping feet to an energetic song, but it can also create muscle relaxation and calmness through entrainment with slower rhythms (14). The oscillatory natures of music and brain waves therefore have been subjects of research not only in neurotypical brains but also in disease states such as anxiety and epilepsy.

### Music and epilepsy

A dichotomous relationship exists between music and epilepsy (15, 16). As a wave-based phenomenon, music can have profound effects on the entire brain starting from its modulatory effect in the brain stem (2, 17). When used correctly, music has the potential to emotionally calm a person with epilepsy (PWEs). On the other hand, when used incorrectly, music can trigger seizures, i.e., musicogenic seizures (18). Seizures themselves can cause musical hallucinations (19).

During the first months of the COVID-19 pandemic, we provided a telemusic intervention program to alleviate the stress and anxiety in patients admitted into the Northwestern Memorial Hospital Neurosciences unit, including PWEs. The program was met with enthusiasm. Patients in the epilepsy monitoring unit (EMU) found the program helpful for the anxiety and distress they experienced during EMU admission. We wondered whether music could be used for emotional modulation within the EMU without interfering with the goals and protocols of EMU testing. Our aim for writing this article is to share our findings during the COVID-19 intervention, and to discuss the possibility of using music as a supportive intervention for patients in the EMU.

### Electroencephalogram as a marker of music-evoked emotions

**Figure 2:**
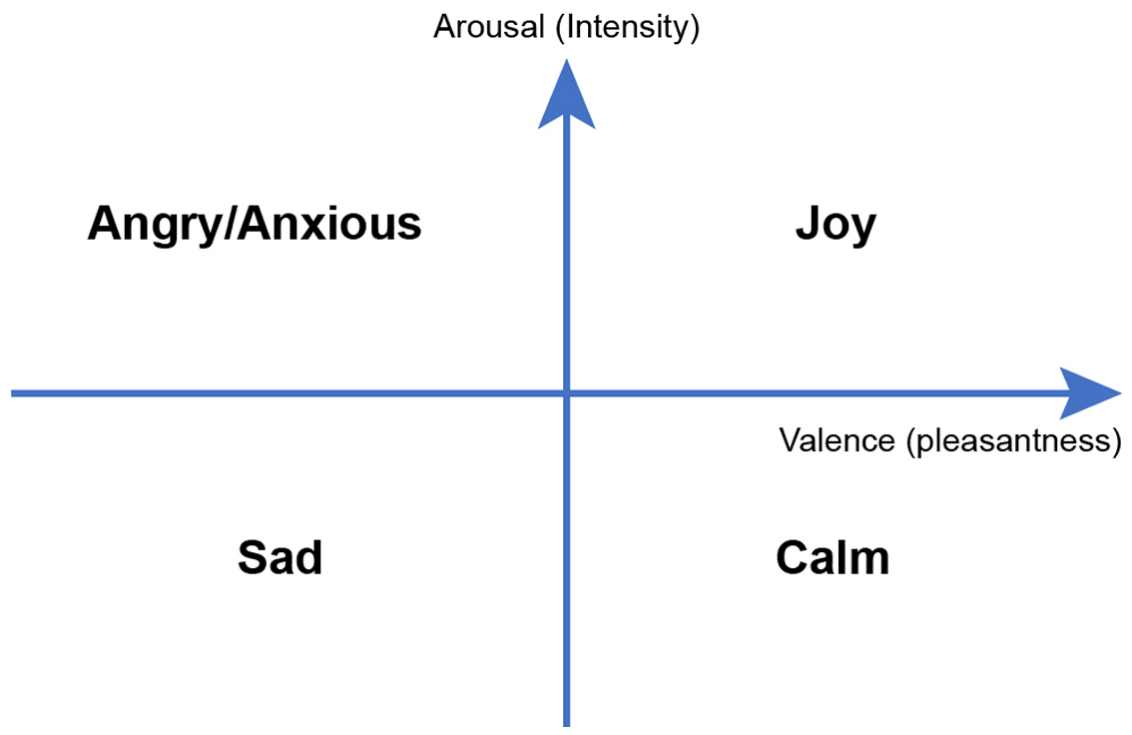
Valence-arousal model of emotions.

Electroencephalogram (EEG) and electrocardiogram (EKG) are readily available in the EMUs and provide an easy way to objectively measure emotional changes. EEG has been endorsed by the National Institutes of Health (NIH) as a reliable way to measure neural activity in response to music interventions (20). A simple way to categorize music-evoked emotions is through the Valence-Arousal model of emotions (Figure 2) where valence ranges from unpleasant to pleasant emotions, and arousal indicates emotional intensity from passive to active. For example, an anxious state contains higher intensity of an unpleasant emotion while calmness is associated with higher intensity of a pleasant emotion (21).

Multiple oscillatory waves can be used to measure music-evoked emotions: *Alpha oscillations* range between 8 and 13 Hertz (Hz) and are believed to be generated in the thalamus where sensory information is gated. There is also evidence that alpha rhythms likely reflect short-range supragranular feedback, which propagate from higher to lower order cortex and from cortex to thalamus (22). Alpha waves represent a low arousal, restful, and relaxed state (23). 10% of individuals with anxiety lack alpha rhythms. *Beta oscillations* are classified in 3 groups: Beta 1 (13–20 Hz), beta 2 (21–30 Hz, also called low gamma), beta 3 (30–60 Hz, also called high gamma), are generated by the basal ganglia and are recorded in the scalp in the Rolandic area. Frontal beta is generated by the frontal cortex. The vertex beta rhythm is induced in response to unexpected situations (24). Beta rhythms are associated with thinking and high arousal (23). *Gamma waves* are associated with increased attention and improved working memory. *Theta oscillations* are between 4 and 8 Hz. The main source of frontal midline theta rhythm is located in the medial prefrontal and anterior cingulate cortex (ACC) (24). High theta band density is associated with calm and low theta density is associated with anxiety. Individuals with anxiety sometimes lack theta rhythms altogether. *Delta waves* originate from the thalamus or the cortex and have a frequency between 0.5 and 4 Hz. Increased delta density is especially important with regard to the depth of sleep cycles and its relationship to immunity, and the sense of restoration after sleep. Delta waves are possibly related to attentional engagement during hours of waking (25).

Higher emotional arousal has been shown to be associated with decreased alpha/beta power (26), while decreased anxiety and increased calmness has been shown to be associated with increased alpha/beta power (27) and decreased delta power (25).

### Discomfort in EMU

Admission to the EMU is to evaluate for seizure frequency, epilepsy, unclear epilepsy syndrome, presurgical evaluation, vertigo, optimizing of therapy, epilepsy characterization, for patients with drug resistant epilepsy, and for individuals who need to be tested for psychogenic non-epileptic seizures (PNES).

The EMU is associated with anxiety due to the altered sense of comfort (e.g., being confined to the bed, lack of sleep, enhanced safety measures) (28). Patients admitted to the EMU are also weaned off their antiseizure medication to enter the EMU, which may have an effect on their neuropsychiatric symptoms (29, 30).

### Mental situation of persons with epilepsy during the COVID-19 pandemic

Similar to other chronic diseases, pandemic isolation and lock down took a significant toll on PWEs. The effect of pandemic precautions on patients’ jobs, low financial status, fear of infection and death by COVID-19, fear of job loss, increased seizures rate during pandemic, increased ER visits, and lack of drug adherence during the pandemic, were significantly associated with increased risk of anxiety and depression. One study reported a rate of anxiety as high as 52.4% in PWEs as compared to a pre-pandemic rate of 20.2% (31, 32). Other studies found depression rates ranging from 39% to 47% in PWEs during the pandemic as compared to a pre-pandemic rate of 22.9% (32, 33). Therefore, PWEs were in high need of care and support during the pandemic.

### Usage of music for EMU patients

The use of art-based interventions in the EMU has been very limited (34). Such interventions would have the potential to support patients to complete their testing if there is a risk of premature termination of EMU testing due to distress and to avoid adverse reactions during EMU admission. Music therapy is less commonly used for adult individuals with epilepsy and further investigations in music therapy for epilepsy are needed. However, there have been reports of using music entertainment for children in pediatric EMUs who have diminished coping and when active engagement is not possible (35).

During the first weeks of the COVID-19 pandemic, strict protocols regarding the presence of family members in the hospitals were instituted causing isolation and anxiety for patients admitted into the neuroscience unit at Northwestern Memorial Hospital. As a general measure, we offered calming Clinically Designed Improvisatory Music (CDIM) to all patients in the unit including EMU patients (36). Post-intervention surveys for all patients in the neuroscience unit were profoundly positive and encouraged us to investigate the EEG responses in these individuals to better understand the underlying mechanisms for their decreased sense of tension and restlessness. We aimed at proving the concept that CDIM is able to induce measurable EEG alterations and generate preliminary evidence as to whether this music intervention can be included as a regular service at the EMU without interfering with EMU protocols.

## Methods

### Participants

CDIM was offered as part of the NMH Telemusic Intervention program during the COVID-19 lockdown to relieve patients’ distress at the NMH Neurosciences unit. During the pilot study, 21 PWEs were identified. Five of these individuals were reported by the nursing and social work staff who interviewed them to have significant distress. The 5 PWEs (4 women and 1 man; average age ± SD = 30.2 ± 6 years) were included in this study to specifically receive CDIM as familiar music alone seemed not to be appropriate for them. Participants’ demographics and diagnoses are listed in Table 1. All consented to listen to the music played through FaceTime and we had permission from our institutional review board (IRB) to retrospectively examine their electroencephalographic changes in response to music.

**Table 1:** Participants’ demographics and diagnoses.

Participant	Age (years)	Gender	Diagnosis
1	33	F	Left frontal focal aware motor and autonomic seizures
2	36	M	Left temporal focal aware dyscognitive seizures; Focal to bilateral tonic–clonic seizures
3	34	F	Frontotemporal focal impaired awareness seizures
4	19	F	Generalized tonic–clonic seizures
5	29	F	Bitemporal (L > R) focal aware seizures

Epilepsy syndromes are reported in accordance with the 2017 International League Against Epilepsy Seizure Classification (ILAE-2107).

### Study design

**Figure 3:**
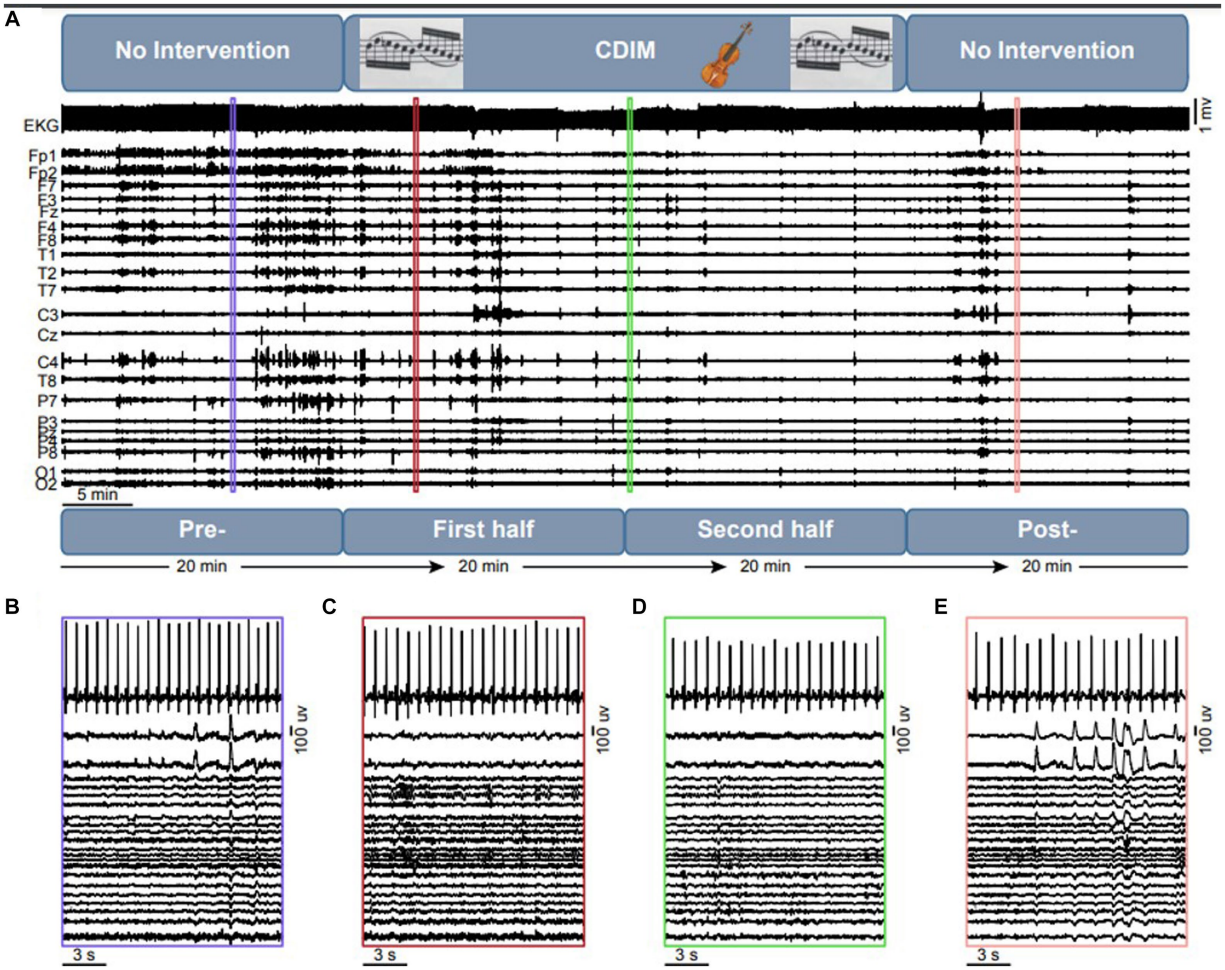
Overview of the intervention design and objective measures. **(A)** The experiment consists of a 20 min no-intervention, which is followed by a 40 min CDIM and 20 min of pos-CDIM no-intervention period. The EKG and EEG data were acquired simultaneously. A 15-s representative segment of EKG and EEG data are shown for the pre-CDIM **(B)**, CDIM1 **(C)**, CDIM2 **(D)** and post-CDIM **(E)** periods.

The study design is summarized in Figure 3. The CDIM intervention lasted 40 min. EEG tracing was analyzed using 4 twenty-minute intervals: 1—pre-CDIM (20 min); 2—CDIM1 (first 20 min); 3—CDIM2 (second 20 min); and 4—post-CDIM (20 min). EEG and EKG were recorded continuously during all intervals with no interruption.

### Music intervention (CDIM)

Patients who were interested in this study consented to participate and filled out the Music Assessment Tool (MAT) (37). In the preliminary section of the MAT, the assessment documented the patient’s name, date, diagnosis, age, level of education, vocation, ethnic background, and religion. This section also documented the reason for admission as well as emotionally significant events prior to admission into the hospital, current mood state, whether the patient’s hearing was impaired, and which musical genres or instruments they liked or disliked.

The certified music practitioner (CMP) presented CDIM virtually and live to enhance the music’s effect on physiologic and social response. Live interaction would allow the CMP to monitor the participant’s body language to enhance improvisation and entrainment. CDIM consisted of 40 minutes of improvisational music avoiding styles the patients disliked preventing adverse reactions to music. The viola was chosen for this research due to its pitch range reflecting the human vocal range. Pitches for the improvised music ranged from 131 Hz to 524 Hz since research shows this range is more likely to stimulate the social engagement network (38) and that it is linked to stimulation of the parasympathetic system (38). CDIM has simple meandering melodies and is played at a slow tempo [50–70 beats per minute (bpm) with an average of 60 bpm] with simple unsyncopated rhythm. The improvisation was slow and meterless, played in the form of 2-minute-long statements ending with a diminuendo which decayed ad nihilum ending in 10 second intervals of silence. After the silence, the musician repeated the same process, improvising under the aforementioned parameters. The start of a new “statement” was not abrupt but in the form of a long soft crescendo. A sample of CDIM is available on the following link: https://www.dropbox.com/scl/fi/8ykk16tz114ny0qzfbly4/CDIM-Edit-15-Min-Cathedral.wav?dl=0&fbclid=IwAR04IjkDdZIn4bnep7RPXMvEGlVO5eTvs7nJXWiOL5LeyhwAkt0ACOdpKQE&rlkey=keywxa9yxdtpgdgnzkq3sp5im.

### Post-CDIM survey

Following the CDIM session, participants responded to a survey to evaluate their experiences. The survey assessed changes in mental and emotional state which participants rated on a Likert scale of 1–10. It consisted of the following items: 1. The music intervention program improved my mental and emotional state. 2. The music intervention program improved feelings of tension. 3. The music intervention program improved feelings of restlessness and/or panic. 4. The music intervention program improved my ability to experience pleasure and contentment. 5. I would recommend this music intervention program to others.

### EEG acquisition and analysis

#### EEG acquisition

The EEG data was collected continuously according to EMU protocol, using a 27-channel Nihon Kohden^©^ (Irvine, CA) data acquisition system Northwestern Memorial Hospital EMU. The international 10–20 system of electrodes placement was used, and the data was recorded at a sampling rate of 1,000 Hz with Cz as the reference. Electrocardiographic data was recorded simultaneously with the EEG data.

#### EEG analysis

The EEG data was band-pass filtered between 0.1 and 100 Hz and re-referenced to a common average reference. Then, the data were down sampled to 200 Hz. The alpha, beta, alpha/beta, theta, delta, and gamma power spectral densities were calculated by condition for each subject. The power spectral density was calculated using Welch’s method with customized MATLAB scripts. First, the EEG data were segmented into continuous 5.12-s epochs (1,024 time points) with an overlap of 90% for each condition, which results in a frequency resolution of 0.195 Hz. To remove epochs with excessive noise, each epoch was *z*-score normalized using the entire time window as baseline. Then the epoch was removed from further analysis if there was any time point in any of the channels that had an absolute *z* score greater than 6. The average number of epochs across all conditions and participants was 1,149 ± 234 (average ± standard error). Then, the spectrum of each epoch was calculated using a fast Fourier transform with a hamming window of 5.12 s. The power spectral density was calculated as the normalized amplitude of the spectrum that is multiplied by its complex conjugate. Finally, the average power spectral density of the delta (0.5–4 Hz), theta (4–8 Hz), alpha (8–13 Hz), beta (13–30 Hz) and gamma (30–100 Hz) frequency bands were calculated, which were normalized by the total spectral density across all frequencies.

The effect of intervention on alpha/beta ratio was evaluated using one-way repeated analysis of variance (ANOVA) for each EEG channel comparing 4-time bins: pre-CDIM intervention (20 min), first half of CDIM intervention (20 min), second half of CDIM intervention (20 min), and post-CDIM intervention (20 min). Post-hoc analysis was performed using paired *t*-test. Due to the low statistical power because of the small sample size of our pilot study, we didn’t correct for multiple comparisons in our analyses.

To estimate the heart rate, the R peaks of the EKG signal were detected using the maximal overlap discrete wavelet transform which is implemented in MATLAB’s Wavelet Toolbox. The heart rate was calculated by dividing the sampling rate, multiplied by 60, by the average R-R duration. To remove artificial R peaks, we detected the R peaks with a minimal distance of 0.15 s (0.5 s for one participant), a threshold that works for our dataset as by eyeballing the result. Then, we calculated the R-R distances, which was further *z*-score normalized. The R-Rs with an absolute z score greater than 1.5 were removed before calculating the heart rate.

To correlate our EEG findings and survey data, we calculated changes in the alpha/beta ratio subtracting pre-CDIM from CDIM and post-CDIM measures. Then, the Pearson correlation coefficient was calculated between the power spectral density changes and survey measures.

## Results

All participants had seizures during their stays and therefore the goal of EMU admission was obtained for all of them. There were no epileptic attacks during the music intervention. Also, there was no evidence of any epileptic attacks 20 min before or after music intervention. Participant 1 had 5 epileptic seizures in the left temporal region during her admission, one of which was before CDIM Participant 2 had one epileptic seizure during his admission before CDIM. Participant 3 had 5 epileptic seizures, one of which was after CDIM. Participant 4 had 4 epileptic seizures, one of which was after CDIM. Participant 5 had 14 epileptic seizures, 3 of which happened after CDIM. Altogether participants had an average (± SD) of 20 ± 3.7 seizures before and 9 ± 1.5 after CDIM. A two-tailed *t* test did not show significant increase or decrease in the number of seizures when number of seizures before and after CDIM were compared (*P* = 0.3).

### Post-CDIM survey

On the post-intervention evaluation, participants reported high ratings for improvement of emotional valence (decreased tension and restlessness, and increased pleasure). They also reported moderate improvement in their energy level. On a 1–10 Likert scale they reported that CDIM resulted in enhanced emotional state at an average of 9 ± 1.26; decrease in tension at 9.6 ± 0.49; decrease in feelings of restlessness at 8.6 ± 0.80; they found the intervention pleasurable at 9.2 ± 0.98. All participants scaled their Likelihood to Recommend (LTR) the program at 10 (Table 2).

**Table 2:** Summary of post-CDIM survey.

Participant	Emotional state	Tension	Restlessness	Pleasure	LTR
1	10	9	8	8	10
2	8	10	8	8	10
3	7	10	9	10	10
4	10	10	10	10	10
5	10	9	8	10	10
Average	9	9.6	8.6	9.2	10
SD	1.26	0.49	0.80	0.98	0

Participants reported their experiences on a Likert scale of 1–10.

### Cardiac tracing

**Figure 4:**
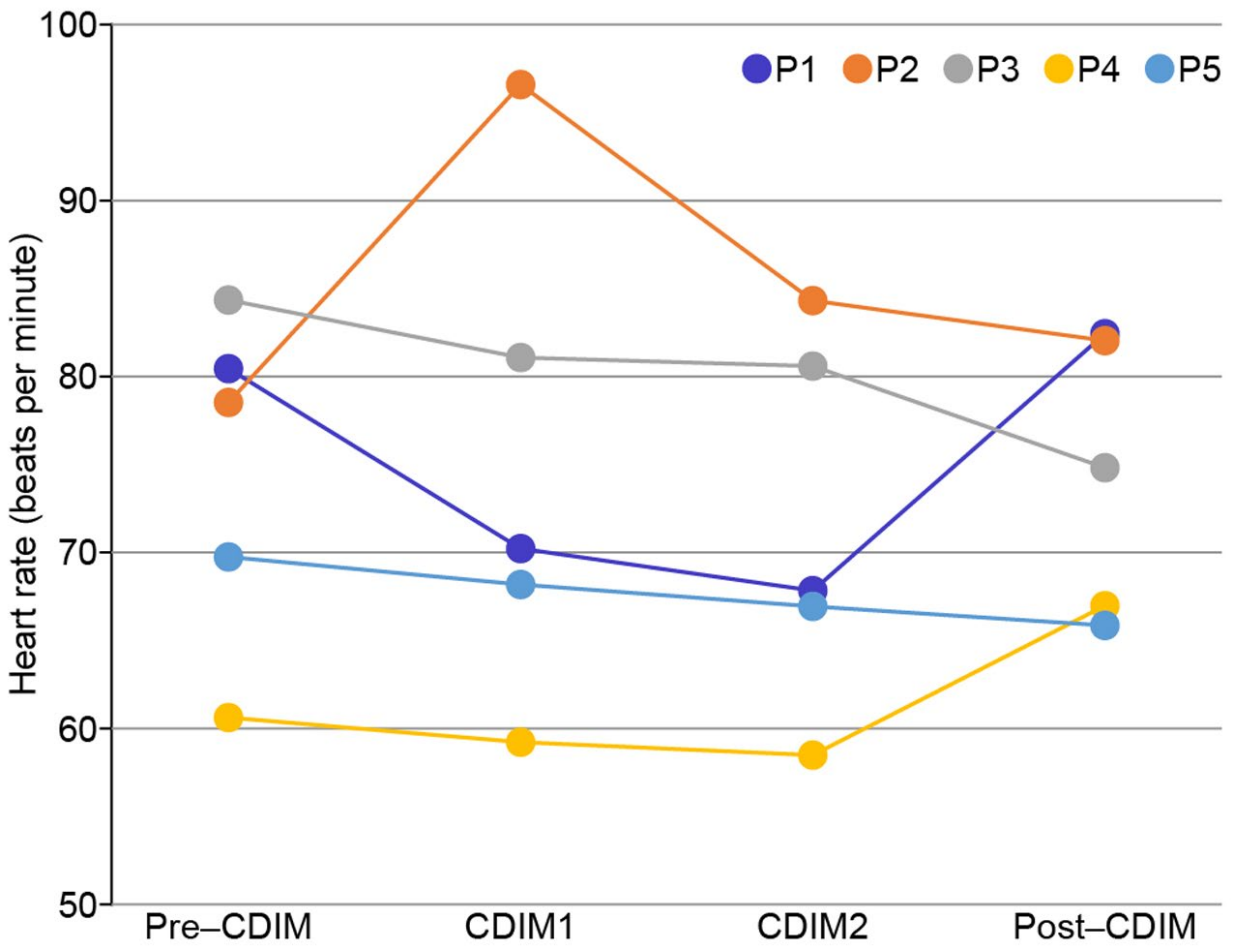
Heart rate changes during CDIM. All participants, except for participant 2 (orange), experienced decreases in heart rate during the intervention.

Cardiac tracing showed a very slight and progressive decrease in heart rate during CDIM1 and CDIM2 interventions for four participants and increased heart rate during CDIM1 followed by a decrease HR during CDIM2 for participant 2 (Figure 4). One-way repeated ANOVA analysis was insignificant for the whole group (F_3, 12_ = 0.37; *P* = 0.78). There was no significant correlation between heart rate and survey measures.

### EEG tracing

#### Whole brain power spectral density analysis for all frequency bands

**Figure 5:**
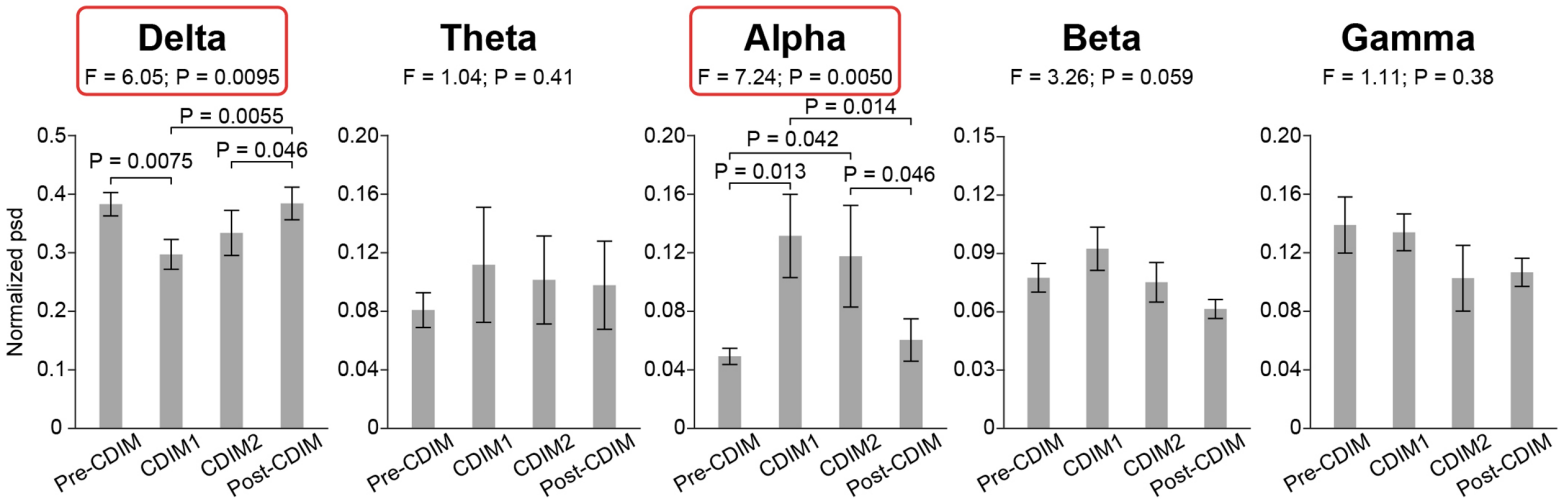
CDIM modulates whole-brain average power spectral density. The whole-brain average normalized power spectral density (psd) was compared across conditions using one-way repeated ANOVA. The bar plot indicates the average normalized power spectral density across participants. The error bar indicates the standard error. Post-hoc test was performed using one-tailed paired *t-*test.

**Figure 6:**
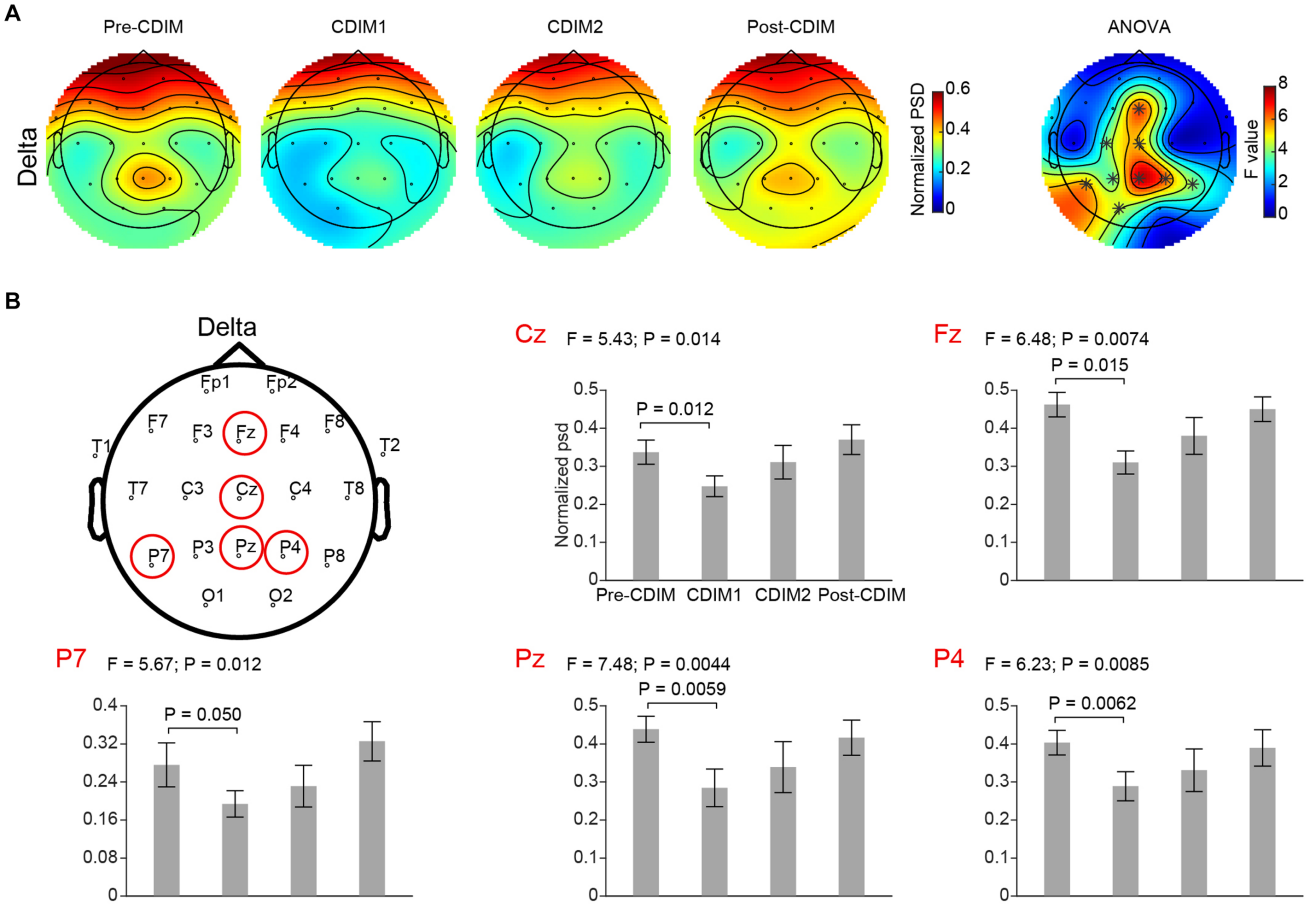
CDIM modulates delta band. **(A)** Topoplot of normalized power spectral density (psd) of the delta frequency band for pre-intervention, during first half of CDIM intervention (CDIM1), during the second half of CDIM (CDIM2) and post-intervention are demonstrated. The right column shows the main effect result from the ANOVA test comparing four conditions. *Indicates uncorrected *P* < 0.05. **(B)** Electrode-based results for the peak regions of the ANOVA test in panel A across four time points. The error bar indicates the standard error. Post-hoc analysis was performed using a one-tailed paired *t*-test.

**Figure 7:**
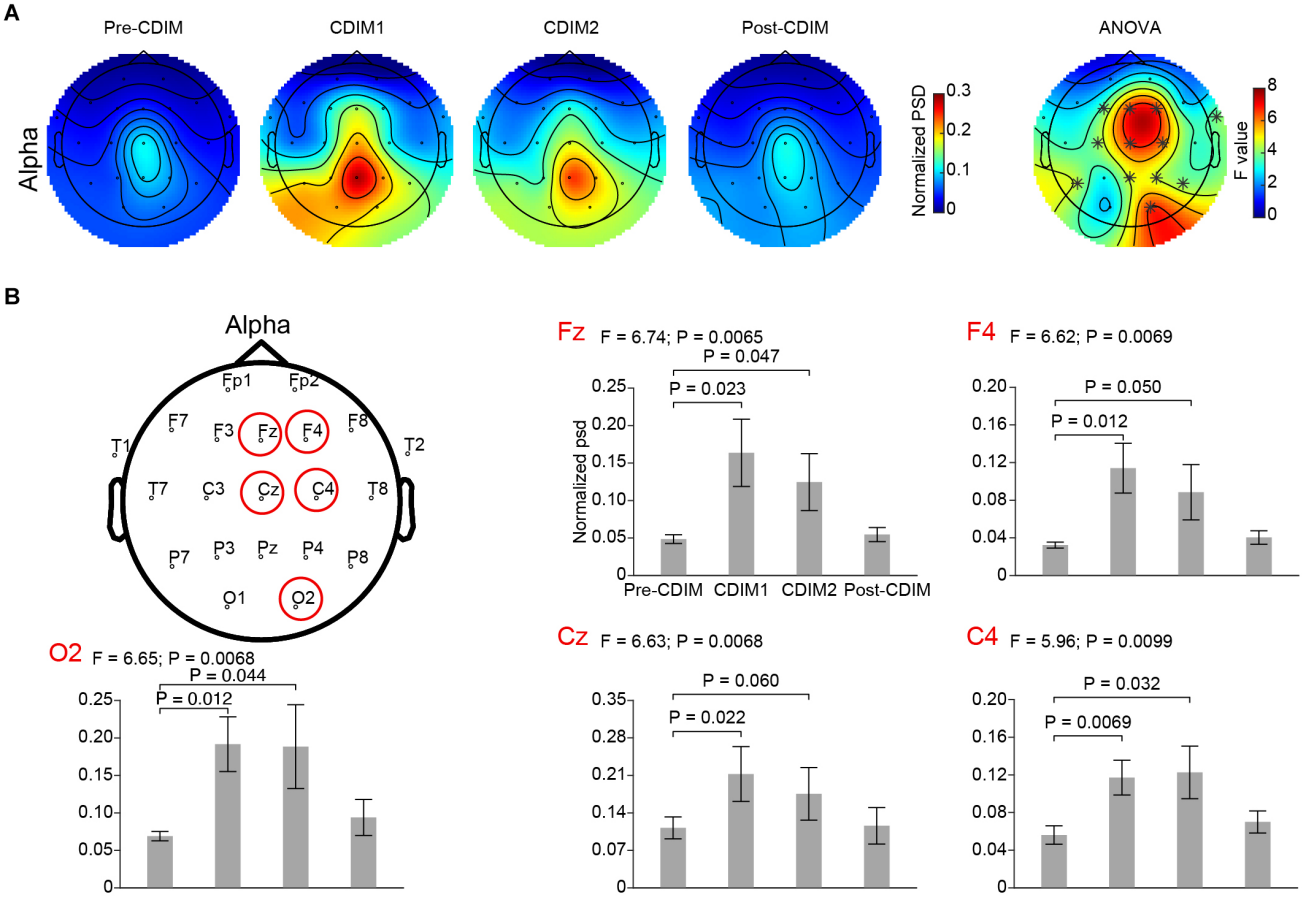
CDIM modulates alpha band. **(A)** Topoplot of normalized spectral density (psd) of the alpha frequency band for pre-intervention, during first half of CDIM intervention (CDIM1), during the second half of CDIM (CDIM2) and post-intervention are demonstrated. Right column shows the main effect result from the ANOVA test comparing four conditions. *Indicates uncorrected *P* < 0.05. **(B)** Electrode-based results for the peak regions of the ANOVA test in panel A across four time points. The error bar indicates the standard error. Post-hoc analysis was performed using a one-tailed paired *t*-test.

To examine the effect of CDIM on power spectral density, we compared the whole-brain average power spectral density across all conditions using one-way repeated ANOVA. We found that CDIM modulates the power spectral density. There was a statistically significant decrease of delta band (F_3, 12_ = 6.05; *P* = 0.0095) and statistically significant increased density in the alpha band (F_3, 12_ = 7.24; *P* = 0.005) during CDIM (Figure 5). No statistically significant effect was found in the theta, beta and gamma frequency band (all *P* > 0.05; Figure 5). Post-hoc analysis suggested a significant effect of CDIM especially during CDIM1 with an increase in alpha band and decrease in delta band densities. The alpha band density trended down after the intervention, however, continued to be higher than baseline after CDIM2 was completed. The opposite was observed for delta waves, with significant decrease during CDIM1 and subsequent return to baseline by post-CDIM interval. Figures 6, 7 illustrates the one-way repeated ANOVA analysis of the delta and alpha power spectral density, respectively, at the single-electrode level.

#### Electrode-based analysis for alpha and delta bands

We performed a one-way repeated ANOVA of the alpha and delta frequency bands for each electrode; the topographic map of the resulting *F* values revealed that the effect for decreased delta band power spectral density was prominent in the midline frontal and parietal regions (Fz, Cz, Pz, P4, and P7; Figure 6A). Post-hoc analysis using paired *t*-test revealed a significant and incremental decrease in delta power spectral density during CDIM compared to pre-CDIM values (Figure 6B). Increase in alpha band power spectral density was prominent in the midline frontal regions (Fz, Cz, F4, and C4; Figure 7A). Post-hoc analysis using paired *t*-test revealed a significant increase in alpha band power spectral density during CDIM compared to pre-CDIM values (Figure 7B).

#### Whole brain analysis for alpha/beta ratio

**Figure 8:**
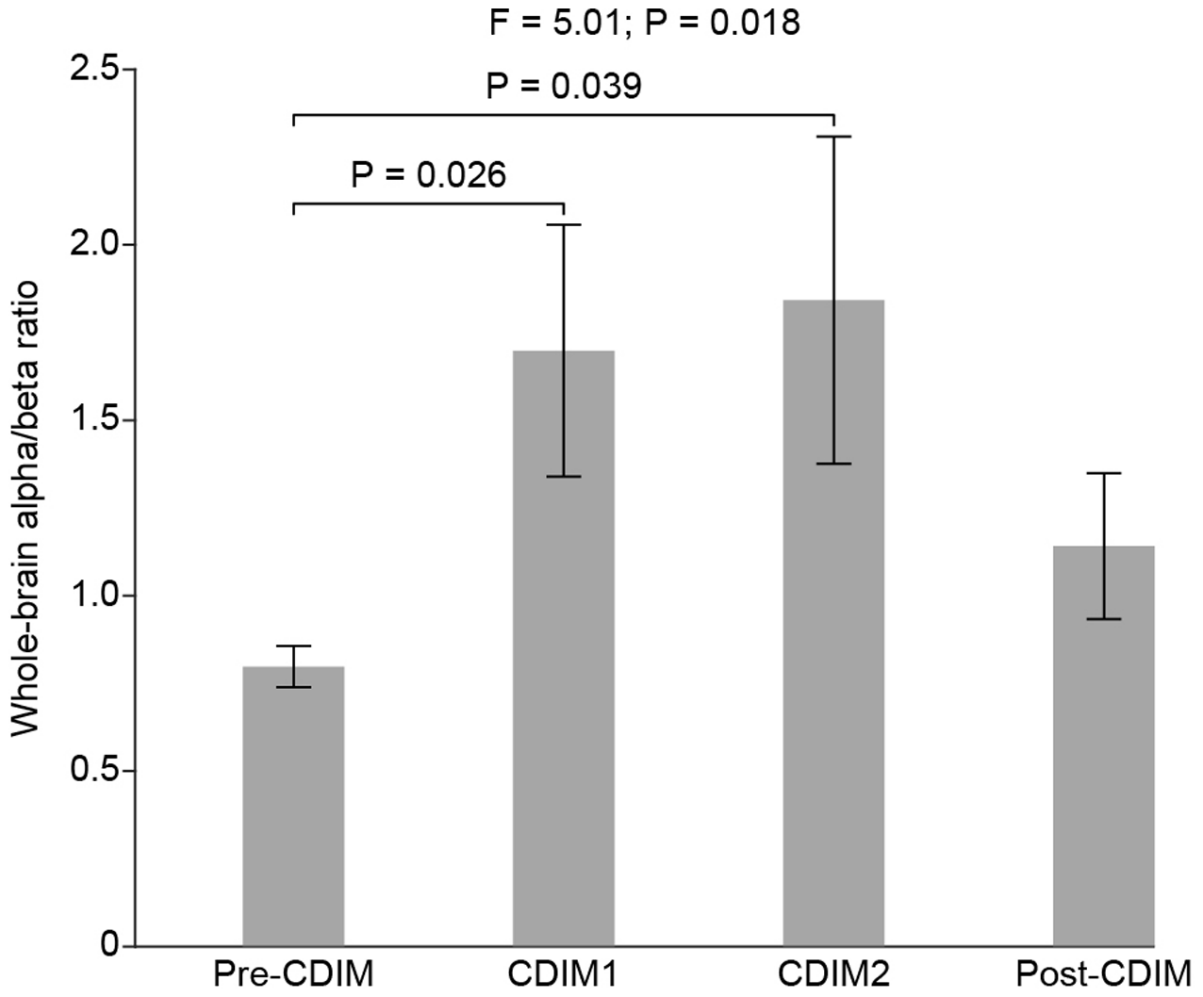
CDIM modulates whole-brain alpha/beta ratio. The whole-brain average alpha/beta ratio was compared across conditions using one-way repeated ANOVA. The bar plot indicates the average alpha/beta ratio across participants. The error bar indicates the standard error. Post-hoc test was performed using a one-tailed paired *t*-test.

To examine the effect of CDIM on alpha/beta ratio, we compared the whole-brain average alpha/beta ratio across all conditions using one-way repeated ANOVA. We found a statistically significant difference in alpha/beta across conditions (F_3, 12_ = 5.01, *P* = 0.018; Figure 8). Post-hoc one-tailed paired *t* test suggested that the alpha/beta ratio was significantly increased for CDIM1 (T_4_ = 2.75, *P* = 0.026) and CDIM2 (T_4_ = 2.36, *P* = 0.039) as compared to the pre-CDIM alpha/beta power (Figure 8).

#### Electrode-based analysis for alpha/beta ratio

**Figure 9:**
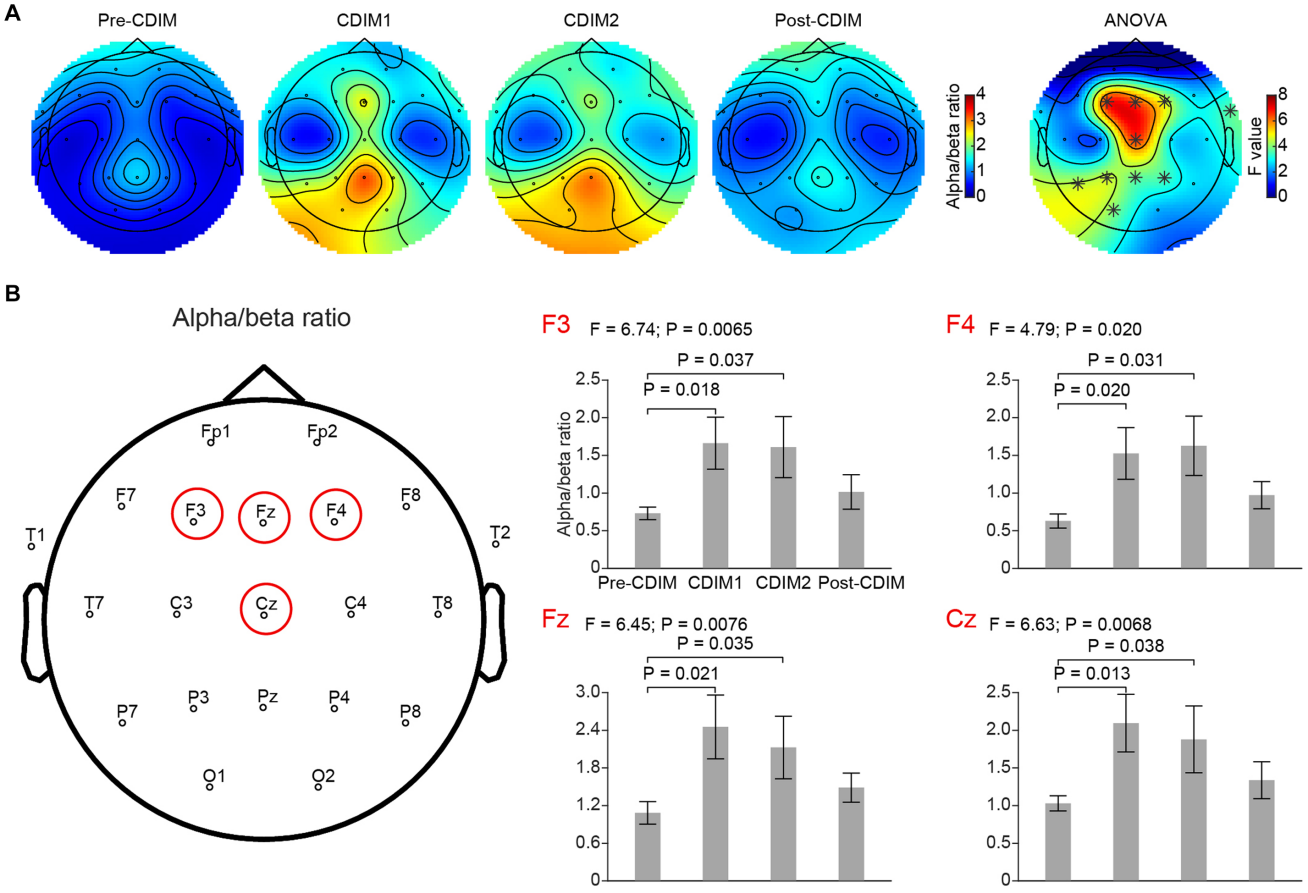
CDIM modulates alpha/beta ratio. **(A)** Topoplot of alpha/beta ratio for pre-intervention (Pre-CDIM), during first half of the CDIM intervention (CDIM1), during the second half of CDIM (CDIM2) and post-intervention (Post-CDIM) are demonstrated. The right column shows the main effect result from the ANOVA test comparing four conditions. *Indicates uncorrected *P* < 0.05. **(B)** Electrode-based results for the peak regions of the ANOVA test in panel A across four time points. The error bar indicates the standard error. Post-hoc analysis was performed using a one-tailed paired *t*-test.

We performed a one-way repeated ANOVA of the alpha/beta ratio for each electrode; the topographic map of the resulting *F* values revealed that the increase in alpha/beta was prominent in the frontal region (F3, Fz, F4 and Cz; Figure 9A). Post-hoc analysis using paired *t*-test revealed a significantly increased alpha/beta ratio during CDIM compared to pre-CDIM values (Figure 9B).

**Figure 10:**
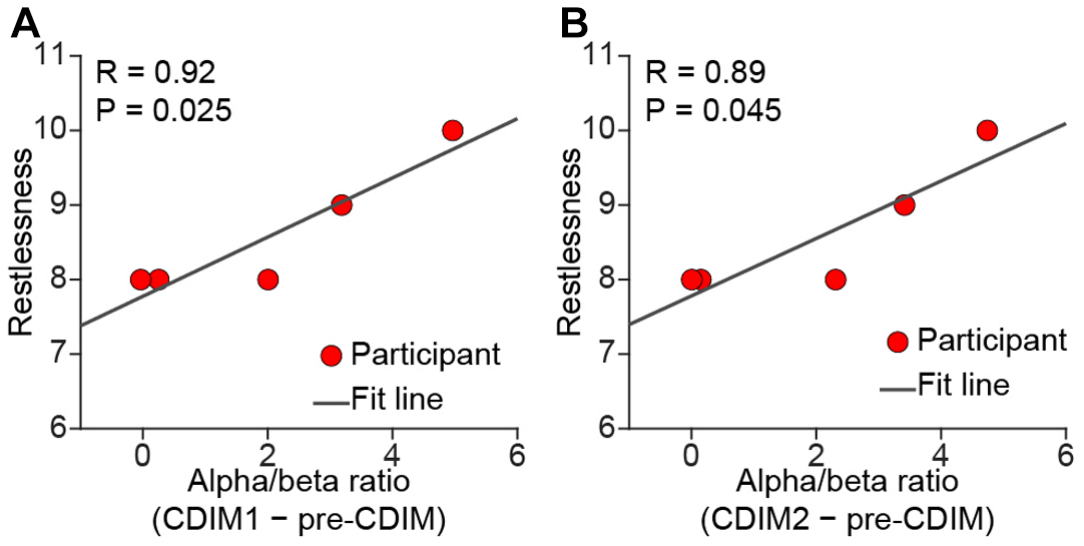
Pearson correlation between alpha/beta and relief from restlessness. There was a positive correlation between alpha/beta and relief from restlessness during both CDIM1 **(A)** and CDIM2 **(B)** phases compared to pre-CDIM, suggesting a positive effect of CDIM.

There was no significant correlation between survey measures and alpha/beta ratio of F3, F4, Fz, and Cz electrodes. However, we found a positive correlation between alpha/beta ratio and decreased restlessness reported from participants in the O1 electrode (Figure 10).

## Discussion

In this pilot study, we demonstrated the feasibility of the CDIM telemusic intervention for five individuals admitted at the NMH EMU during the COVID19 lockdown. Participants agreed at a rate of ≥90%, with an improvement in their emotional valence, decrease in symptoms related to tension, and found the experience pleasurable. Objectively, they had a significant decrease in the delta band density with peaks at midline, right dorsal parietal, and left posterior temporal regions, an increase of the alpha band density with peaks at frontal midline and right fronto-central regions, and an increase in the alpha/beta ratio with peak at frontal midline and dorsal prefrontal frontal regions. Relief from restlessness positively correlated with spectral density of alpha/beta ratio in the O1 occipital electrode.

### Effect of CDIM on stress

#### Physiologic effect of CDIM

Decreased heart rate and levels of stress in response to calming music has repeatedly been shown in the literature (39). In our study we found a decrease in average heart rate during CDIM intervention, however, statistically insignificant, likely due to lack of power. In four out of five participants, however, heart rate had a downward trend. All patients agreed with the subjective decreased stress level.

#### Effect on stress and EEG correlate

On EEG analysis, the CDIM intervention was associated with an increase in alpha, and decrease in delta band power spectral density. Decreased midline delta is likely related to engagement with the music and heightened internal attention reported in successful meditation studies in the absence of drowsiness (25). This effect was observed mostly during the first half of CDIM intervention. Progressive increases in midline frontal alpha power signify a change from an aroused to a less aroused, more restful, and relaxed state (23).

Increased alpha/beta ratio is in line with previous studies reporting decreased anxiety in individuals with psychiatric disorders and also in EEG research on meditative states (27, 40). The peak of increased alpha/beta in this study was located at Fz, Cz (frontal midline/anterior cingulate regions), and F3, F4 (bilateral prefrontal regions) which are regions all reported to be engaged in meditative states (40, 41). Clinical observation of focused attention to music decreased stress-driven movements and decreased heart rates during the CDIM in four out of five patients, and significant changes in this region may suggest a state of less distraction, less brain noise, and more calmness. This role is likely exerted through the auditory limbic (emotional modulation) network being entrained to the slow rhythm of CDIM. Slow rhythms and tempi induce a feeling of calmness emotionally and through descending efferent pathways modulate the autonomic nervous system through parasympathetic activation (42).

While peak regions of increased alpha/beta ratio were mostly symmetrical, changes in alpha and delta waves peaked at midline and in the right hemisphere, likely due to the right lateralized effect of music on emotions (43). In case of changes in the delta wave, there was a peak at the left posterior temporal region likely related to parsing CDIM “statements” interspersed by pauses (44). Altogether, the above electroencephalographic changes prove the concept that CDIM engages the brain in expected regions of the brain that are involved in listening to music, mindfulness, and musical structure.

We were not able to show a direct correlation between survey measures and F3, Fz, F4, and Cz electrodes. However, relief from restlessness positively correlated with alpha/beta density within the O1 electrode during both CDIM1 and CDIM2. Research on alpha oscillations has shown a striking propagation of alpha traveling from anterosuperior cortex toward posteroinferior areas. It is possible that we are capturing the terminus of such propagation. Figure 9A may help understanding this phenomenon where there seems to be an anterior-posterior gradient of alpha/beta ratio during CDIM1 and CDIM2.

### Clinical utilization of CDIM in the EMU setting

Music as a therapeutic tool is underutilized in medical care in the United States. Music therapists and practitioners are yet to find a way to be reimbursed for their services since many states lack legislation approving insurance coverage for such services (45). In a previous study (36) we demonstrated how a group of 80 patients admitted in the neuroscience unit at NMH benefited from the telemusic intervention as “psychological first aid” during the COVID-19 lock-down. The telemusic intervention was therefore found to be feasible. The EMU patients included in the current study received CDIM as part of the same intervention. As they were already connected to EEG, we were able to objectively study their physiological and brain responses to the 40-minute music intervention. This created an opportunity to consider the utility of using music for the EMU.

It may seem contradictory to use an intervention that brings relief to EMU patients when the aim of the EMU is to provoke seizures through controlled stressors. However, the reason to seriously consider an intervention such as CDIM for the EMU is that approximately 7%–12% of EMU patients cannot complete the EMU testing because the stress involved in order to provoke seizures is overwhelming (46). In addition, due to the stringent safety protocols required in the EMU, patients are subject to extreme limitations for what they can do during their 2–7 day hospitalization. We propose that music interventions such as CDIM be used when there is increased irritability and risk of discontinuation on the patient’s side, since CDIM did not interfere with the aims of EMU testing but provided significant relief to the patient. CDIM can also be used at the end of the patient’s EMU stay as a restorative measure during the time they are getting back on their medications. Musicogenic seizures generally are evoked by strong memory associations with particular pieces of music or songs due to sensitization (15). For this reason we believe CDIM would be safe for EMU patients because unfamiliar and improvisatory content would be unlikely to spark strong associations and thus would avoid causing musicogenic seizures. While this is a small study, we did not find any harm associated with the administration of CDIM. When the number of seizures before and after CDIM were compared, there was no significant increase in the number of seizures.

## Limitations

Our study has obvious limitations and only serves as a proof of concept. We report results in only five patients which does not allow for generalizations. Furthermore, we examined the post-CDIM 20 min right after CDIM intervention; the long-term effect of CDIM on the alpha/beta ratio and power spectral density changes observed in this study needs to be further evaluated. The lack of behavioral survey before CDIM, as well as other clinical outcome of patients such as seizures, epileptiform discharges, EEG seizure patterns, quality of life, intelligence quotient, stigma, adherence, changes in medication (reduction of anti-seizure medication to record seizures) and physiological state (awake/sleep, time interval from means, and any other stimulus that can cause EEG and heart rate changes), prevents us from statistically evaluating the effect of CDIM on these parameters. In our future studies, we will include these parameters as we expand this line of research.

While we had “internal” control phases (pre- and post-CDIM recordings), results would be more reliable when a separate control group were to be used for comparison. We plan to evaluate these findings for individuals with no epileptic activities in the future. We also plan to expand this study using a randomized controlled trial with two groups of patients admitted to the EMU with one group receiving the CDIM and another one receiving a placebo intervention. In this way we can improve our current knowledge based on the NIH staged model for behavioral interventions (47, 48).

## Data availability statement

The raw data supporting the conclusions of this article will be made available by the authors, without undue reservation.

## Ethics statement

The studies involving human participants were reviewed and approved by Northwestern University Institutional Review Board. The patients/participants provided their written informed consent to participate in this study.

## Author contributions

BB: literature search, study design, interpretation, and writing the entire manuscript. GZ, DH, and CZ: analysis of EEG data, interpretation, and writing the results section. CV: literature search and writing the background section. SS: interpretation of results and all clinical aspects of the study. CT: clinician musician, literature search, study design, interpretation, and writing the entire manuscript. All authors contributed to the article and approved the submitted version.

## Funding

This study was supported by Northwestern Department of Neurology Philanthropy (BB) and R01-DC-018539 (CZ).

## Conflict of interest

The authors declare that the research was conducted in the absence of any commercial or financial relationships that could be construed as a potential conflict of interest.

## Publisher’s note

All claims expressed in this article are solely those of the authors and do not necessarily represent those of their affiliated organizations, or those of the publisher, the editors and the reviewers. Any product that may be evaluated in this article, or claim that may be made by its manufacturer, is not guaranteed or endorsed by the publisher.
